# Elevated neurofilament light levels in acute anorexia nervosa are associated with alterations in white matter volume and connectivity networks

**DOI:** 10.1111/jcpp.70083

**Published:** 2025-12-02

**Authors:** Inger Hellerhoff, Daniel Geisler, Fabio Bernardoni, Arne Doose, Friederike I. Tam, David M. Poitz, Nina Chotjewitz, Veit Roessner, Katja Akgün, Tjalf Ziemssen, Stefan Ehrlich

**Affiliations:** ^1^ Division of Psychological and Social Medicine and Developmental Neuroscience, Faculty of Medicine Technische Universität Dresden Dresden Germany; ^2^ Institute of Clinical Chemistry and Laboratory Medicine University Hospital Carl Gustav Carus, Technische Universität Dresden Dresden Germany; ^3^ Department of Child and Adolescent Psychiatry, Faculty of Medicine University Hospital Carl Gustav Carus, Technische Universität Dresden Dresden Germany; ^4^ Center of Clinical Neuroscience, Neurological Clinic, Faculty of Medicine University Hospital Carl Gustav Carus, Technische Universität Dresden Dresden Germany; ^5^ Department of Neurology Sächsisches Krankenhaus Arnsdorf Arnsdorf Germany; ^6^ Translational Developmental Neuroscience Section, Eating Disorder Research and Treatment Center, Department of Child and Adolescent Psychiatry, Faculty of Medicine Technische Universität Dresden Dresden Germany

**Keywords:** Anorexia nervosa, neurofilament light, white matter volume, white matter connectivity, leptin

## Abstract

**Background:**

Anorexia nervosa (AN) is a severe eating disorder associated with drastic reductions in gray and white matter (WM) volume and structural connectivity alterations. However, the hypotheses regarding underlying mechanisms are inconclusive. The current study investigated the relationships of WM volume as well as WM network architecture with neurofilament light (NF‐L), a marker of axonal damage.

**Methods:**

Blood samples and magnetic resonance imaging scans from 77 predominantly adolescent female participants with acute AN were used. Associations of WM volume with NF‐L were tested using linear models. The relationship between NF‐L and alterations in brain networks was evaluated using network‐based statistic (NBS) models, which predicted connectivity associated with NF‐L levels. Additionally, associations with clinical variables and leptin were tested. To test the specificity of the results, control analyses were conducted on 77 female healthy participants (HC).

**Results:**

We found negative associations between NF‐L concentrations and WM volume. NBS analyses identified seven components, where fractional anisotropy was positively associated with NF‐L. In some components, mean connectivity was negatively associated with leptin concentrations. Mediation analyses suggested that the negative correlation of leptin and NF‐L might be partially mediated by changes in WM microstructure. These effects were not observed in HC.

**Conclusions:**

The results suggest that WM volume reductions in acute AN might be related to axonal damage. The NBS results indicate, that the elevated fractional anisotropy previously found in AN might be related to damage processes leading to axonal swelling. All in all, the present study supports NF‐L as a global blood marker for brain damage processes in acute AN.

## Introduction

Anorexia Nervosa (AN) is a serious eating disorder that typically begins during adolescence (American Psychiatric Association, 2013). It is characterized by a persistent restriction in energy intake leading to severe medical complications (Voderholzer, Haas, Correll, & Körner, 2020; Westmoreland, Krantz, & Mehler, 2016). Brain structure is substantially altered in acute AN, including changes in white matter (WM), structural connectivity, and gray matter (GM; Bahnsen et al., 2022; Geisler et al., 2022; Seitz, Konrad, & Herpertz‐Dahlmann, 2018).

Several studies in large datasets have shown that AN goes along with substantial volume reductions in GM and WM, and cortical thinning (Asami et al., 2022; Bahnsen et al., 2022; Cascino et al., 2020; Seitz et al., 2018). These seem to be mainly related to undernutrition and the resulting low body weight (Bernardoni et al., 2016). However, the pathomechanisms underlying these changes remain unclear (King, Frank, Thompson, & Ehrlich, 2018). Studies investigating protein markers of brain damage in participants with AN suggest that damage processes to neuronal axons and astrocytes might contribute to the structural alterations (Hellerhoff et al., 2021; Nilsson et al., 2019; Wentz et al., 2021). In a recent study, higher baseline serum levels of the protein neurofilament light (NF‐L) in young participants with acute AN, were associated with lower cortical thickness (CT) in several brain regions (Hellerhoff et al., 2023). NF‐L is a structural scaffolding protein (Khalil et al., 2018) and increased cerebrospinal fluid (CSF) or blood concentrations of NF‐L are indicative of neuroaxonal injury (Gaetani et al., 2019; Khalil et al., 2024). Associations with WM volume or connectivity would therefore be expected as well.

WM microstructure has also shown to be affected in AN, with findings of mainly decreased WM integrity (Barona et al., 2019; Frank, Shott, Hagman, & Yang, 2013; Laczkovics et al., 2022; Meneguzzo, Collantoni, Solmi, Tenconi, & Favaro, 2019; Nickel et al., 2019). However, some studies focusing on WM connectivity have also found increased connectivity in AN (Pfuhl et al., 2016; Vogel et al., 2016). This might be due to starvation‐related factors, such as axonal swelling (Vogel et al., 2016) due to damage processes, leading to higher fractional anisotropy (FA). A recent study (Geisler et al., 2022) used a network‐based statistic (NBS) method to identify WM networks characterized by altered connectivity in AN. Six WM subnetworks were identified, in which increased FA indicated abnormal network architecture. Possible explanations for these findings include starvation‐related factors such as increased density in axonal packing or premorbid factors (Geisler et al., 2022). Concurrently, the study found three components of decreased number of streamlines (NOS) in AN. Investigations of NF‐L might help understand the processes underlying these seemingly contradictory findings. If starvation‐related damage processes were the underlying cause of subnetworks with higher FA, these findings should be accompanied by elevated NF‐L concentrations. For the purposes of this study, we describe WM connectivity as the structural connections between brain regions that are mediated by WM fiber tracts. FA and NOS are used as a proxy for WM connectivity. We estimate WM connections based on FA/NOS of the tractography‐derived streamlines and summarized in a connectivity matrix, reflecting the strength of interregional connections. The NBS approach is then used to identify connectivity patterns that differ across groups.

Additionally, the study explores the potential relationship between WM alterations in AN and hypoleptinemia. Leptin is an adipocyte‐derived hormone showing a strong positive correlation with fat mass in addition to BMI and acting as a signal to the brain regarding the availability of energy resources (Considine et al., 1996; Hebebrand, Muller, Holtkamp, & Herpertz‐Dahlmann, 2007; Liu, Yang, Yu, & Zheng, 2018). Hypoleptinemia is a central feature of AN (Hebebrand et al., 2007) and recent studies have found links between local volumetric reductions in the amygdala and the hippocampus in AN and lower leptin levels (Bahnsen et al., 2024; Wronski et al., 2022). Associations of leptin levels and WM changes in AN have not yet been investigated but would be expected due to the involvement of leptin in processes such as neurogenesis, axon growth, and synaptogenesis (Paz‐Filho, Wong, & Licinio, 2010).

The aim of the present study was to inquire how NF‐L can provide insight into possible WM damage processes in acute AN. We wanted to test whether the protein is associated with disease‐related changes in WM structure and WM network architecture.

For this purpose, two sets of analyses were conducted:

1. Standard analyses testing whether elevated levels of NF‐L are related to decreased measures of WM volume in acute AN.
2. NBS analyses to test the association of NF‐L with brain network alterations in acute AN.

Further analyses were conducted to gain insights into the interplay between brain structural alterations, serum NF‐L and clinical features of acute AN such as hypoleptinemia.

## Methods

### Participants

Diffusion weighted imaging (DWI) scans and NF‐L measurements were available from 80 underweight young (median age 15.7 years), female participants with acute AN, diagnosed according to the Diagnostic and Statistical Manual of Mental Disorders (DSM‐5; American Psychiatric Association, 2013). 114 healthy control participants (HC) were available for supplementary analyses. As this was a follow‐up study to Geisler et al. (2022), we used the participants who had passed quality control (QC) of the DWI data and age matching in that study. Availability of blood measurements restricted the final DWI sample to 77 participants with AN and 77 HC and the sMRI sample to 74 participants with AN and 74 HC (due to some datasets not being usable for in‐depth structural analyses; Supporting Information Appendix S1, sections 1.1 and 1.4.2 for details). Participants with AN were studied in the acutely underweight state shortly (mean: 2.92 days) after admission to intensive treatment. Data was collected between 2011 and 2017. The study was approved by the ethics committee of the Technische Universität Dresden (protocol numbers EK 536122015/EK 39022012) and all participants (and the legal guardians of underage participants) gave written informed consent.

AN was diagnosed using a modified version of the German expert form of the Structured Interview for Anorexia and Bulimia Nervosa (SIAB‐EX; Fichter & Quadflieg, 1999) and required a BMI < 17.5 kg/m^2^ (or below the 10th age percentile, if <15.5 years). HC had to be of normal weight, eumenorrhoeic, and without any psychiatric illness (assessed by the Mini International Neuropsychiatric Interview (Sheehan et al., 1998, 2010)). Additional exclusion criteria were applied to AN and HC participants, including psychotropic medication in the four weeks preceding study participation (except for selective serotonin reuptake inhibitors, allowed in AN participants, *n* = 3; Appendix S1, section 1.1).

### Clinical Measures

Psychopathology was assessed using the SIAB‐EX (Fichter & Quadflieg, 1999), the subscales ‘drive for thinness’ and ‘body dissatisfaction’ of the German version of the Eating Disorder Inventory‐2 (EDI‐2; Paul & Thiel, 2005), and the German version of the Beck Depression Inventory‐II (BDI‐II; Hautzinger, Keller, & Kühner, 2009). IQ was estimated using age‐appropriate versions of the Wechsler Intelligence Scales (Appendix S1, section 1.2). Age‐ and gender‐corrected BMI standard deviation scores (BMI‐SDS; Hemmelmann, Brose, Vens, Hebebrand, & Ziegler, 2010; Kromeyer‐Hauschild et al., 2001) were calculated from the BMI.

### Blood Sampling and Analysis

Blood sampling was carried out as previously described (Appendix S1, section 1.3 Hellerhoff et al., 2021; Tam et al., 2022) after an overnight fast. NF‐L levels were determined using the digital Simoa™ Human Neurology 4‐Plex A assay in combination with the Simoa™ HD‐1 Analyzer or the digital Simoa® NF‐light™ Advantage Kit in combination with the Simoa™ HD‐X Analyzer (all Quanterix, Lexington, MA, USA) following the manufacturer's instructions (Appendix S1, section 1.3; Doose et al., 2021; Hellerhoff et al., 2021).

Blood for leptin, was collected into EDTA vacutainer tubes (Sarstedt, Nümbrecht, Germany). Plasma leptin was measured using an enzyme‐linked immunosorbent assay (BioVendor, Brno, Czech Republic). Leptin concentrations below the lower limit of detection (LOD = 0.20 ng/ml; *n* = 17 AN and 0 HC) were imputed (Appendix S1, section 1.3). Missing/unavailable values (2 AN and 2 HC) were not imputed.

### MRI Acquisition and Processing

MRI scanning took place between 8 and 9 a.m. following an overnight fast. High‐resolution three‐dimensional T1‐weighted structural scans were acquired on a 3 T scanner (Magnetom Trio, Siemens, Erlangen, Germany) using a rapid acquisition gradient echo (MP‐RAGE) sequence with the same parameters as previously reported (Appendix S1, section 1.4.1; Bahnsen et al., 2022; Bernardoni et al., 2016).

DWI data were collected using a spin‐echo sequence at 2.4 mm isotropic voxel resolution with the technical parameters described in previous studies (Geisler et al., 2022; Appendix S1, section 1.4.1). A total of 32 diffusion sensitizing gradients (*b* = 1,300 s/mm^2^) were applied, and 4 images without diffusion weighting (*b* = 0 s/mm^2^) were acquired.

#### Processing of Structural MRI

##### WM Volume Estimation

We used standard FreeSurfer (version 5.3) segmentation procedures to extract measures of WM volume in 73 regions of interest (ROI; Dale, Fischl, & Sereno, 1999; Desikan et al., 2006; Fischl, Sereno, & Dale, 1999; Fischl et al., 2002, 2004; Fischl & Dale, 2000). A complete list of the parcellations is provided in Appendix S1, section 1.4.2 and Table S1. The quality of the segmentation was assured by visual inspection by a trained examiner with the support of quality assurance tools implemented in FreeSurfer. Participants not meeting quality criteria were excluded a priori from the analyses.

#### Processing of DWI

##### Preprocessing

The DWI preprocessing pipeline has been described in detail previously (Van den Heuvel, Mandl, Stam, Kahn, & Hulshoff Pol, 2010), and is described in the Appendix S1, section 1.4.2.

##### Fiber Tracking

Using the extracted information on the preferred diffusion direction within each voxel in the brain mask, the WM tracts of the whole brain network were reconstructed using the deterministic fiber tracking (Van den Heuvel et al., 2010), based on the fiber assignment by continuous tracking (FACT) algorithm (Mori, Crain, Chacko, & Van Zijl, 1999). Fibers were reconstructed by starting 8 seeds in each voxel and following the main diffusion direction of each voxel (selected as the principal eigenvector) until one of the following stopping criteria was reached: the fiber tract entered a voxel with a low level of diffusion preference (FA < 0.1), made an unexpected sharp angular turn (angle > 45°), or left the brain mask.

##### Network Construction

For network construction (see below), the T1 image of each participant was segmented into 233 regions of interest (ROIs; 219 cortical and 14 subcortical) on the basis of a high‐resolution subdivision of FreeSurfer's Desikan‐Killiany atlas (Cammoun et al., 2012). Further details regarding the preprocessing of the data and the atlas are provided in Appendix S1, section 1.4.2. We refer to the 233 ROIs as nodes, which are pairwise connected by edges associated with weights. For every edge, we determined fibers crossing the 2 given ROIs. The NOS of an edge is the count of fibers associated with the edge. The FA value of an edge is the respective weighted mean of all voxels crossed by the fiber segments connecting the ROIs (Hagmann et al., 2010; Van den Heuvel et al., 2010). The network construction resulted in 2 symmetric 233 × 233 connectivity matrices per participant: FA‐weighted and NOS‐weighted.

##### Quality Control

For each participant, missing edges and outliers were determined as described in Geisler et al. (2022; details in Appendix S1, section 1.4.2).

### Statistical Analyses

#### Clinical Variables and NF‐L Levels

Due to violations of normal distribution, NF‐L concentration values were log‐transformed or robust statistical tests were used (see Table 1). Extreme outliers (>3 standard deviations from the mean of the respective diagnostic group) were excluded from all analyses (*n* = 1 AN and *n* = 1 HC).

**Table 1:** Demographic and clinical characteristics of the study sample

	AN^a^		HC		AN vs. HC^d^		
	Median [IQR]	*n*	Median [IQR]	*n*	*W*/*t*	*p*	*r*
Age	15.70 [14.30; 17.20]	77	17.00 [15.10; 18.50]	77	2,308.5	.018*	0.191
IQ	115.00 [106.00; 124.00]	70	110.00 [104.00; 117.00]	76	3,347.5	.007**	0.223
BMI	14.77 [13.94; 15.71]	77	20.35 [19.56; 21.86]	77	35	<.001***	0.853
BMI‐SDS	−2.73 [−3.64; −2.22]	77	−0.18 [−0.61; 0.27]	77	0	<.001***	0.863
Minimal lifetime BMI	14.52 [13.55; 15.24]	77	19.54 [18.32; 20.88]	72	35	<.001***	0.852
EDI‐2 ‘Drive for thinness’	30 [22; 36]	75	13 [8; 16]	76	5,135	<.001***	0.693
EDI‐2 ‘Body dissatisfaction’	37 [28.75; 45.00]	76	20 [16; 26]	76	4,833	<.001***	0.582
BDI‐II	21.00 [15.00; 30.00]	77	3.00 [1.00; 7.00]	76	5,636.5	<.001***	0.800
NF‐L (pg/ml)^b^	10.80 [7.83; 15.50]	77	5.98 [4.73; 7.79]	77	8.3662	<.001***	0.586
Leptin (ng/ml)^c^	0.58 [0.165; 1.635]	75	10.4 [5.895; 15.035]	75	201	<.001***	0.801

AN, participants with acute anorexia nervosa; HC, healthy control participants; W, Test statistic of the Wilcoxon rank sum test with continuity correction; r, Effect size of the group comparison (Wilcoxon effect size r for the Wilcoxon rank sum test and Pearson correlation for the t‐Test); BMI, body mass index; BMI‐SDS, body mass index standard deviation score; EDI‐2, Eating Disorder Inventory, version 2; BDI‐II, Beck Depression Inventory, version 2; *p*‐values < .05 were considered statistically significant and all significant group differences remained significant when applying false discovery rate correction for multiple comparisons (correction for 10 group comparisons of descriptive variables and marker values). *FDR < .05, **FDR < .01, ***FDR < .001.

^a^ 69 participants with AN were of the restrictive and 8 of the binge‐eating/purging subtype. 13 participants with AN were diagnosed with one or more active comorbid psychiatric disorders (5 with depression, 3 with obsessive‐compulsive disorder, 2 with anxiety disorders, 2 with tic disorders 1 with adaptation disorder, 1 with mutism, and 1 with somatoform disorder).

^b^ For better interpretability, median and IQR of the raw marker values are displayed. However, group comparisons were computed with log‐transformed marker values due to slight violations of normal distribution (all comparisons one‐sided due to prior hypotheses except for age and IQ).

^c^ For leptin, all calculations were done using the imputed values (see methods section).

^d^ Group differences for demographic variables were examined using nonparametric Wilcoxon rank sum tests and differences for log‐transformed NF‐L levels using Welch's t‐tests (details in Appendix S1, section 1.5.1).

#### WM Volume

Associations of WM volume with NF‐L in AN were examined using linear models predicting WM volume from NF‐L, age, and estimated total intracranial volume (eTIV; the latter two as control variables). FDR correction was applied to account for multiple comparisons in all WM analyses (correction over 73 tests). To test whether the effects found are specific to AN, the model was also applied to a HC group.

Supplementary analyses were performed to test for group differences in WM volume (Appendix S1, section 1.5.2 and Appendix S2, section 2.6).

##### Relationship to Clinical Variables

To explore associations between WM volume and clinical variables (‘Drive for Thinness’, ‘Body Dissatisfaction’, BDI‐II total, leptin, duration of illness, BMI‐SDS), robust regression analyses were conducted for total cortical WM volume as well as for the left and right hemispheres separately. Predictors included the clinical variables and age as a control variable (Appendix S1, section 1.5.3).

#### DWI – Network‐Based Statistic

NBS is a powerful statistical method for identifying a statistically significant cluster of connections (also called a component) influenced by a specified explanatory variable (Zalesky, Fornito, & Bullmore, 2010).

To examine the relationship between NF‐L and brain network alterations in individuals with AN, we assessed two NBS models, predicting connectivity (FA and log‐transformed NOS) in relation to NF‐L levels. FA is a widely used metric in clinical and translational diffusion MRI research and may reflect aspects of WM microstructure, but it is important to acknowledge that it does not directly represent anatomical connectivity strength.

As described in our previous studies (Ehrlich et al., 2015; Geisler et al., 2019), NBS are computed using the following steps: (i) identify all edges that exhibit modulation by the explanatory variable (in a linear model with edge weight as outcome) beyond a predefined t‐value (primary threshold), (ii) select all contiguous clusters of these modulated edges, and (iii) validate the cluster's significance by permutation testing. In permutation testing, an empirical null distribution of the largest cluster size is generated by conducting the first two NBS steps on data with the explanatory variable permuted 10,000 times. The returned subnetworks are statistically significant at a family‐wise error‐controlled threshold of *p* < .05.

The NBS procedure was carried out for NOS and FA matrices with a primary threshold of *t* = 3.2 (corresponding to *p* = .001). To account for potential confounding effects of WM volume, motion, and age, these metrics were included as covariates. To test whether the effects found are specific to AN, we also applied the model to a HC group.

To confirm the result, NBS was carried out across a range of primary thresholds. FA results remained robust across different primary thresholds, whereas NOS results did so to a lesser extent (Appendix S2, section 2.4 and Figure S1).

##### Relationship to Clinical Variables

The mean connectivity values for components with a significant association to NF‐L in AN were calculated for each participant by averaging the participant's weights of all edges included in a given component. To explore associations with clinical variables in AN, we fitted robust linear models (Appendix S1, section 1.5.3). The predictor variables tested included ‘drive for thinness’, ‘body dissatisfaction’, depressive symptoms, leptin, duration of illness, and BMI‐SDS. All regression models included age, WM volume, and motion as nuisance variables.

We extracted the standardized regression coefficients of the clinical variables and estimated the *p*‐values using Wald tests. We adjusted for multiple comparisons using the FDR method across components (7 tests). Additionally, we performed robust mediation analyses using bootstrapping methods to test whether the correlation seen between leptin and NF‐L concentrations could be statistically accounted for by connectivity values. Although not causal, this approach is increasingly used in neuroimaging to explore potential mechanistic pathways (Adhikari et al., 2022; Ajilore et al., 2015; Xu & Kang, 2023).

#### Sensitivity Analyses on the Influence of Antidepressant Medication or AN Subtype

Sensitivity analyses investigating the potential influence of antidepressant medication and subtype are reported in the (Appendix S1, section 1.5.4 and Appendix S2 section 2.1 and Table S2).

#### Software

Demographic and clinical variables, damage marker levels, WM volume, and relationships between connectivity networks/WM volume and clinical variables were analyzed using R (Appendix S1, section 1.5.6); (R Core Team, 2023).

## Results

### Study Sample and Clinical Measures

Demographic and clinical characteristics of the study sample are summarized in Table 1. Participants with AN had significantly lower BMI‐SDS and minimal lifetime BMI and higher symptom levels than HC (EDI‐2 and BDI‐2). Participants with AN were also slightly, but significantly younger than HC and had a significantly higher IQ. As expected, NF‐L levels were significantly elevated in participants with AN compared to HC and leptin levels were significantly lower in AN than in HC. In AN, NF‐L was negatively correlated with BMI, BMI‐SDS, minimal lifetime BMI, and leptin concentrations (complete correlation matrix in Table S3). The correlation of NF‐L and leptin remained significant when controlling for BMI‐SDS (Appendix S2, section 2.3). In HC, no correlations were seen.

### Association of NF‐L Levels with WM Volume

Global measures of WM volume were negatively associated with NF‐L in AN (higher NF‐L associated with lower WM volume) but not in HC (Table 2, Figure S2). Of the local parcellations, the fusiform, paracentral, precentral and superior frontal areas of the left hemisphere and the caudal middle frontal, fusiform, inferior temporal, precuneus and superior frontal areas of the right hemisphere were negatively associated with NF‐L in AN after correction for multiple testing (complete table including nonsignificant regions in Table S4). In HC, only the left cuneus was associated with NF‐L in the opposite direction to the effects seen in AN. The group differences between AN and HC seen in some local measures of WM volume did not survive FDR correction for multiple comparisons (Appendix S2, section 2.6, Table S5). WM volume (total, left hemisphere, and right hemisphere) in AN was not associated with any of the clinical variables tested.

**Table 2:** Associations of NF‐L levels with white matter volume

	AN			HC		
	β	Stand. β	*p*	β	Stand. β	*p*
Left hemisphere						
Cortical white matter volume	−24,338.787	−0.277	<.001**	20,009.114	0.136	.041
Cuneus	−164.449	−0.097	.407	1,012.326	0.382	<.001*
Fusiform	−851.282	−0.247	.005*	−185.288	−0.034	.706
Paracentral	−661.972	−0.313	.005*	119.555	0.038	.713
Precentral	−1,989.726	−0.302	.003*	1,269.966	0.145	.109
Superior frontal	−2,285.745	−0.288	.002*	1,300.336	0.100	.245
Right hemisphere						
Cortical white matter volume	−23,306.922	−0.258	<.001**	20,529.462	0.141	.035
Caudal middle frontal	−1,328.280	−0.330	.002*	671.888	0.122	.244
Fusiform	−1,052.605	−0.269	<.001*	−177.839	−0.032	.719
Inferior temporal	−930.195	−0.267	.007*	−156.425	−0.036	.706
Precuneus	−1,382.181	−0.250	.008*	1,320.467	0.138	.123
Superior frontal	−1,792.241	−0.221	.006*	1,560.577	0.109	.175
Global						
Cortical white matter volume	−47,645.709	−0.269	<.001**	40,538.576	0.138	.036

The table shows the estimates (β) and *p*‐values for neurofilament light (NF‐L) as a predictor in the regressions predicting the denoted WM volume measures (controlling for age and estimated total intracranial volume). For better readability, only regions where NF‐L significantly predicted WM volume are displayed (full table in SI). WM volume is indicated in mm^3^. The asterisks denote the following significance levels after false discovery rate (FDR) correction for 73 tests (including nonsignificant regions): *FDR < .05, **FDR < .01.

### Association of NF‐L with WM Connectivity Networks in AN

**Figure 1:**
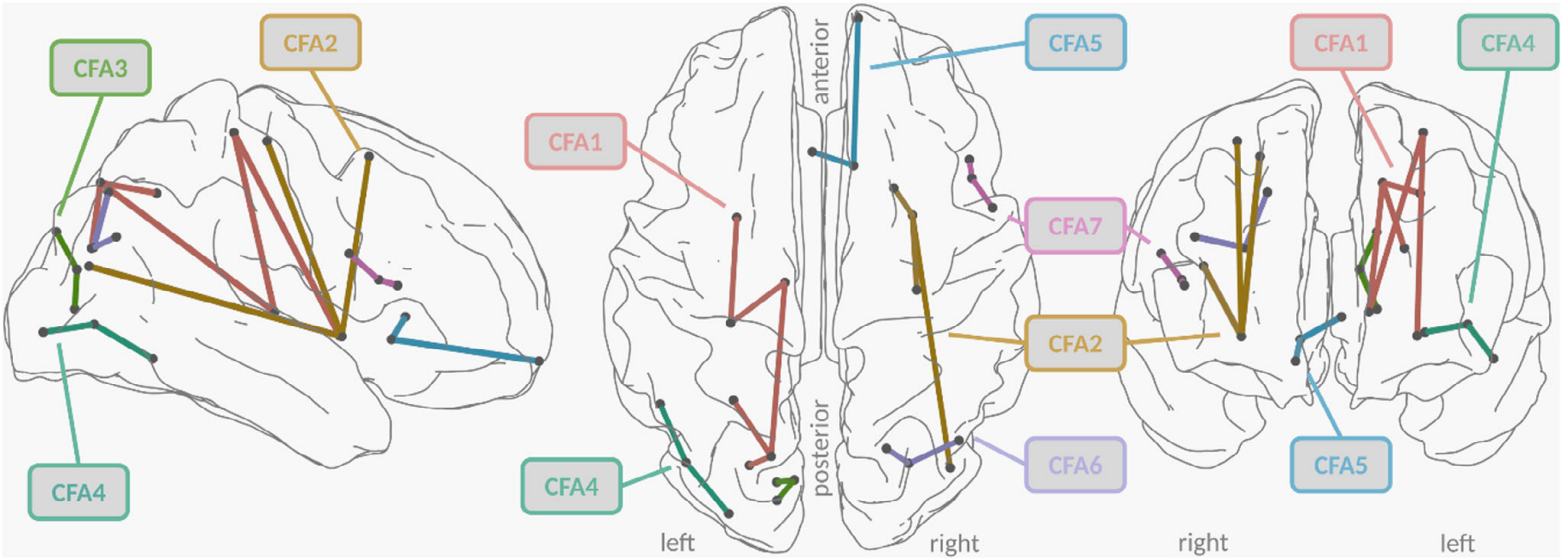
Network‐based statistic (NBS) components where NF‐L was associated with FA. Results of the NBS at the primary threshold of *t* = 3.2 are shown. Components in which higher neurofilament light (NF‐L) was associated with higher fractional anisotropy (FA) in participants with anorexia nervosa (AN) are depicted as connected edges of the same color

We found seven distinct components, where FA was positively associated with NF‐L (Figure 1). The largest component (CFA1) comprised 5 edges spanning over the superior parietal and postcentral regions of the left hemisphere, left thalamus and left putamen. The second largest component (CFA2) comprised inferior parietal, precentral and superior frontal regions of the right hemisphere and the right putamen. The smaller components CFA3 to CFA7 are described in Table 3.

**Table 3:** Connections of fractional anisotropy (FA) network‐based statistic (NBS) components where FA was predicted by NF‐L

**Component CFA1: LH Thalamus‐superior parietal connections (𝑝 = .0012)**	
Left‐Thalamus‐Proper Left‐Thalamus‐Proper Left‐Putamen ctx‐lh‐superiorparietal_5 ctx‐lh‐superiorparietal_3	– ctx‐lh‐postcentral_2 – ctx‐lh‐superiorparietal_5 – ctx‐lh‐postcentral_2 – ctx‐lh‐superiorparietal_6 – ctx‐lh‐superiorparietal_5
**Component CFA2: RH Putamen connections** (*p* = .0076)	
Right‐Putamen Right‐Putamen Right‐Putamen	– ctx‐rh‐inferiorparietal_6 – ctx‐rh‐precentral_5 – ctx‐rh‐superiorfrontal_8
**Component CFA3: LH Cuneus connections** (*p* = .038)	
ctx‐lh‐cuneus_1 ctx‐lh‐cuneus_1	– ctx‐lh‐pericalcarine_1 – ctx‐lh‐superiorparietal_7
**Component CFA4: LH Lateral occipital connections** (*p* = .038)	
ctx‐lh‐inferiortemporal_4 ctx‐lh‐lateraloccipital_3	– ctx‐lh‐lateraloccipital_4 – ctx‐lh‐lateraloccipital_4
**Component CFA5: Rostral anterior cingulate connections** (*p* = .038)	
ctx‐rh‐rostralanteriorcingulate_1 ctx‐lh‐rostralanteriorcingulate_1	– ctx‐rh‐frontalpole_1 – ctx‐rh‐rostralanteriorcingulate_1
**Component CFA6: RH Superior parietal connections** (*p* = .038)	
ctx‐rh‐inferiorparietal_4 ctx‐rh‐superiorparietal_5	– ctx‐rh‐superiorparietal_6 – ctx‐rh‐superiorparietal_6
**Component CFA7: RH Pars opercularis connections** (*p* = .038)	
ctx‐rh‐parsopercularis_1 ctx‐rh‐parsopercularis_2	– ctx‐rh‐parsopercularis_2 – ctx‐rh‐precentral_2

Components (with their respective edges) where NF‐L significantly predicted FA at the primary threshold of *t* = 3.2. Edges are listed as pairs of ROIs. Also refer to Figure 1. FA, fractional anisotropy; lh, left hemisphere; NBS, network‐based statistic; rh, right hemisphere.

At the threshold of *t* = 3.2, no components, in which NF‐L significantly predicted the logarithmized NOS, were found.

### No Association of NF‐L with FA Connectivity Networks in HC

In HC, no network components, in which NF‐L was associated with FA, were identified. In one very small component of two edges in the right hemisphere (caudal anterior cingulate and superior frontal regions), NF‐L was associated with the NOS (Figure S3).

### Associations between Illness‐related Variables and NBS Components in AN

In AN, associations between mean connectivity in the components of the main model, where NF‐L was associated with FA and leptin, were seen (components 1, 2, 3, 5, and 6, Table 4). These negative associations also remained significant when controlling for BMI‐SDS (Table S6). No associations for other illness‐related variables were found (Table 4). A secondary analysis excluding imputed leptin values confirmed that the result was robust (Table S7).

**Table 4:** Complete table of associations between mean connectivity in the components and clinical variables

Predictor	Component	β	*p*
BMI‐SDS	CFA1	−0.115	.441
BMI‐SDS	CFA2	−0.165	.215
BMI‐SDS	CFA3	−0.321	.029
BMI‐SDS	CFA4	−0.082	.561
BMI‐SDS	CFA5	−0.022	.763
BMI‐SDS	CFA6	−0.243	.044
BMI‐SDS	CFA7	−0.140	.340
Duration of illness	CFA1	0.084	.587
Duration of illness	CFA2	0.139	.308
Duration of illness	CFA3	0.079	.614
Duration of illness	CFA4	0.113	.391
Duration of illness	CFA5	−0.092	.217
Duration of illness	CFA6	0.054	.672
Duration of illness	CFA7	−0.072	.632
Leptin	CFA1	−0.349	.005**
Leptin	CFA2	−0.310	.007**
Leptin	CFA3	−0.345	.004**
Leptin	CFA4	−0.147	.215
Leptin	CFA5	−0.254	<.001**
Leptin	CFA6	−0.361	<.001**
Leptin	CFA7	−0.234	.052
BDI‐II	CFA1	−0.014	.909
BDI‐II	CFA2	−0.165	.177
BDI‐II	CFA3	−0.122	.328
BDI‐II	CFA4	0.116	.325
BDI‐II	CFA5	0.0468	.490
BDI‐II	CFA6	−0.138	.203
BDI‐II	CFA7	0.041	.747
EDI‐2 ‘Drive for thinness’	CFA1	0.102	.438
EDI‐2 ‘Drive for thinness’	CFA2	−0.063	.604
EDI‐2 ‘Drive for thinness’	CFA3	−0.056	.659
EDI‐2 ‘Drive for thinness’	CFA4	0.127	.300
EDI‐2 ‘Drive for thinness’	CFA5	0.033	.636
EDI‐2 ‘Drive for thinness’	CFA6	−0.188	.082
EDI‐2 ‘Drive for thinness’	CFA7	−0.082	.541
EDI‐2 ‘Body dissatisfaction’	CFA1	0.091	.474
EDI‐2 ‘Body dissatisfaction’	CFA2	−0.097	.410
EDI‐2 ‘Body dissatisfaction’	CFA3	0.056	.647
EDI‐2 ‘Body dissatisfaction’	CFA4	0.185	.120
EDI‐2 ‘Body dissatisfaction’	CFA5	0.013	.842
EDI‐2 ‘Body dissatisfaction’	CFA6	−0.146	.182
EDI‐2 ‘Body dissatisfaction’	CFA7	−0.058	.659

The table shows the estimates (β) and *p*‐values for illness‐related variables as predictors in the robust regression models predicting mean connectivity in components with a significant association with neurofilament light (NF‐L) in anorexia nervosa. BMI‐SDS, body mass index standard deviation score; EDI‐2, Eating Disorder Inventory, version 2; BDI‐II, Beck Depression Inventory, version 2. The asterisks denote significance levels after false discovery rate (FDR) correction (7 tests per variable): **FDR < .01.

**Figure 2:**
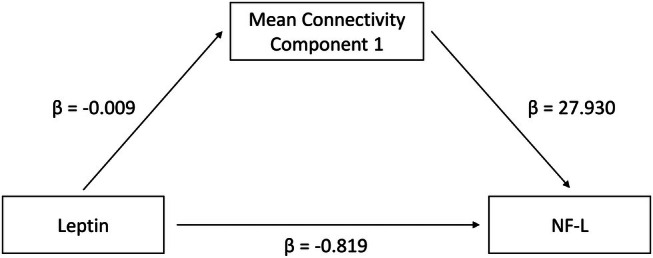
Exemplary illustration of the mediation analysis results for component CFA1. Regression coefficients for the direct and indirect effect of leptin concentrations on neurofilament light (NF‐L) concentrations are shown. Regarding significance of the indirect effect in the six components see Table 5

Mediation analyses in the components, where leptin concentrations were significantly associated with mean connectivity, showed that in components 1, 3, and 6 the association of leptin concentrations with NF‐L was partially mediated via the (altered) mean connectivity (Table 5 and Figure 2). This effect remained significant when applying FDR correction over the five tests performed.

**Table 5:** Results of mediation analyses in the components where leptin concentrations were significantly associated with mean connectivity

	Estimate	*z*‐value	*p*	95% Confidence interval	
				Lower	Upper
Component CFA1					
Direct effect: leptin → NF‐L	−0.819	−4.031	<0.001	−1.245	−0.430
Indirect effect: leptin → connectivity → NF‐L	−0.238		0.014*	−0.608	−0.041
Total effect: leptin → NF‐L	−1.057	−4.721	<0.001	−1.520	−0.628
Component CFA2					
Direct effect: leptin → NF‐L	−0.848	−4.183	<0.001	−1.247	−0.451
Indirect effect: leptin → connectivity → NF‐L	−0.218		0.101	−0.849	0.032
Total effect: leptin → NF‐L	−1.065	−4.138	<0.001	−1.601	−0.572
Component CFA3					
Direct effect: leptin → NF‐L	−0.652	−3.529	<0.001	−1.039	−0.297
Indirect effect: leptin → connectivity → NF‐L	−0.354		0.002**	−0.765	−0.127
Total effect: leptin → NF‐L	−1.006	−5.184	<0.001	−1.411	−0.637
Component CFA5					
Direct effect: leptin → NF‐L	−0.906	−2.633	0.008	−1.720	−0.252
Indirect effect: leptin → connectivity → NF‐L	−0.045		0.385	−0.215	0.080
Total effect: leptin → NF‐L	−0.951	−3.027	0.002	−1.699	−0.363
Component CFA6					
Direct effect: leptin → NF‐L	−0.819	−3.996	<0.001	−1.248	−0.427
Indirect effect: leptin → connectivity → NF‐L	−0.217		0.021*	−0.625	−0.028
Total effect: leptin → NF‐L	−1.031	−4.439	<0.001	−1.531	−0.593

Estimates, (*z*‐values), *p*‐values, and the lower and upper bounds of the 95% confidence interval are shown for the direct, indirect, and total effects in the mediation analyses for each component. False discovery rate (FDR) correction was applied to the *p*‐values of the indirect effects, to account for 6 components tested. Significance of the other effects was not a subject of the analysis. Asterisks indicate the following significance levels after FDR correction: *FDR < .05, **FDR < .01.

## Discussion

We tested whether alterations in the neuronal damage marker NF‐L are associated with disease‐related changes in WM structure and network architecture in a predominantly adolescent sample of participants with acute AN. We found negative associations between NF‐L concentrations and global as well as local measures of WM volume. When analyzing the network structure in AN, we found seven components, where FA was positively associated with NF‐L, suggesting damage processes underlying elevated FA values in AN. In three of these components, mean connectivity was also negatively associated with leptin concentrations. Correlations between NF‐L and leptin concentrations and additional mediation analyses suggest that the negative association of leptin concentrations with NF‐L might be partially mediated by WM microstructure changes reflected by elevated FA.

Brain structural alterations in AN are well studied and manifest as reductions in GM and WM volume, reduced CT and alterations in WM microstructure and connectivity (Asami et al., 2022; Bahnsen et al., 2022; Barona et al., 2019; Cascino et al., 2020; Frank et al., 2013; Geisler et al., 2022; Seitz et al., 2018). It has been hypothesized, that brain structural alterations in AN stem at least partially from ongoing damage processes to brain cells (Hellerhoff et al., 2021, 2023; Nilsson et al., 2019). These could include morphological changes to dendrites as well as soma (King et al., 2018; Neumärker et al., 1997; Seitz et al., 2018). This notion is supported by a recent rodent study, which found decreased brain volume to be associated with increased NF‐L, decreased microglia density, increased soma area, and prolonged microglial processes (Zimmermann et al., 2025). The present first finding of a negative association between WM volume and NF‐L in AN in humans aligns with the assumption that NF‐L is a marker for axonal damage (Gaetani et al., 2019; Khalil et al., 2024). In the context of other psychiatric disorders, elevated concentrations of NF‐L have previously been inversely associated with WM volume in alcohol dependence (Li et al., 2021) and longitudinally with WM atrophy especially in cognitively impaired individuals (Benedet et al., 2020). When parcellating the WM, associations were found in two main clusters: the first cluster comprises inferior temporal and fusiform areas. These areas overlap with brain areas, in which we previously found an association between increased NF‐L and reduced CT as a measure of GM atrophy (Hellerhoff et al., 2023). The inferior temporal cortex and the fusiform gyrus are involved in visual tasks including body perception and might be related to the body image distortion often seen in AN (Conway, 2018; Weiner & Zilles, 2016). The second cluster comprised superior frontal, precentral, paracentral, and caudal middle frontal regions. These are regions, in which CT reductions are usually less pronounced in acute AN (Bahnsen et al., 2022). NF‐L is abundant in large‐caliber myelinated axons (Gaetani et al., 2019; Zetterberg, Smith, & Blennow, 2013). Speculatively, longer myelinated axons connecting different brain regions, might be affected in these regions even if the GM volume is not substantially altered. Importantly, research suggests, that in adolescence, typical remodeling of the brain cortex is accompanied by the formation of short‐distance intracortical myelin fibers. These natural developmental processes may interfere with disruptions caused by AN (Corrigan et al., 2021).

The second part of the study focused on associations of NF‐L with WM connectivity networks in AN. The finding of seven components, in which NF‐L positively predicted FA, might seem counterintuitive at first, since elevated NF‐L levels could be expected to be associated with lower FA. However, our results are in line with the study by Geisler et al. (2022) on the same patient population, who found components of increased FA in participants with AN compared to HC. Two other studies support this finding: Vogel et al. (2016) also found increased FA in AN and Frank, Shott, Riederer, and Pryor (2016) found increased WM connectivity between specific regions. A hypothetical explanation of these findings is that starvation‐related factors such as axonal swelling due to damage processes might be at the origin of higher FA values in AN (Geisler et al., 2022; Vogel et al., 2016). Our results of elevated NF‐L (as a marker of axonal damage) associated with elevated FA support this hypothesis (Gaetani et al., 2019). Especially the fact that these effects occurred alongside an association between WM volume reductions and NF‐L, suggests that the elevated FA in AN might actually be a sign of WM microstructural damage.

The biggest component in which NF‐L was positively associated with FA in AN (CFA1), was mainly composed of connections of the thalamus to superior parietal and postcentral regions. Previous studies have found decreased thalamus connectivity in AN (Geisler et al., 2016; Hayes et al., 2015), which aligns with our findings suggesting microstructural damage to these connections. The second component (CFA2) comprised connections of the putamen with other brain regions. The putamen is part of the striatum and the cortico‐striatal‐thalamic circuit involved in reward learning and motivation (Chau, Roth, & Green, 2004). Energy restriction and the resulting weight loss are thought to alter the dopamine‐ventral striatal reward system, reinforcing self‐starvation possibly by attributing motivational value to cues associated with food restriction (Sala, Egbert, Lavender, & Goldschmidt, 2018; Zink & Weinberger, 2010). However, any functional implications of our results can only be speculative.

Hypoleptinemia as a consequence of undernutrition might contribute to brain structural alterations in AN (Bahnsen et al., 2024). In the present study, we found an association between leptin and NF‐L in participants with AN but not in HC. Leptin concentrations were also negatively associated with mean FA in the network model in AN and in three components, the association of leptin concentrations with NF‐L was partially mediated by the (altered) mean FA. We speculatively hypothesized that hypoleptinemia might lead to damaged WM connectivity, resulting in an increased release of NF‐L, as seen in the associations between increased NF‐L and increased FA in participants with AN. Due to the study design, this mechanistic hypothesis has to be regarded as purely speculative, but it is in line with recent findings of cross‐sectional and longitudinal associations between leptin and GM volume in AN (Bahnsen et al., 2024; Wronski et al., 2022, 2024). Given that WM is composed of the sheathed nerve processes of the GM, there may well be a connection in WM as well. This is further supported by studies reporting negative associations of leptin with WM integrity in depression and schizophrenia (Amer et al., 2019; Wang et al., 2020).

Our results have to be considered in the light of some limitations. The study focused on a female adolescent sample and thus might not be generalizable to adult and more chronic as well as male patients. Second, the present results in patients with AN do not allow us to clearly differentiate between effects of starvation (which might be present also in individuals undernourished for other reasons) and psychological effects of AN. Third, pubertal status was not assessed in the study sample. Fourth, we measured NF‐L in blood samples. While blood–brain barrier integrity may theoretically affect NF‐L levels, correlations between blood and CSF levels of NF‐L have been demonstrated in various neurological conditions, including those involving WM damage or neuroinflammation (Alagaratnam et al., 2021; Hansson et al., 2017; Hofmann et al., 2024; Khalil et al., 2024). Fifth, future studies should use more recent FreeSurfer versions. Last, a further limitation of this study is the use of FA as a connectivity weight. FA could be influenced by multiple factors such as axonal density, myelination, and fiber coherence, and may not adequately capture complex fiber configurations like crossings, potentially limiting its specificity.

To summarize, we used a multimethod approach to shed light on mechanisms underlying WM structural alterations in AN. We found evidence that axonal damage processes might be a reason for WM volume reduction as well as counterintuitive neuroimaging‐based structural connectivity changes seen in AN. Together with previously reported associations between elevated NF‐L concentrations and reduced CT in participants with AN, these results also show that NF‐L might be used to monitor brain structural changes occurring on multiple levels in AN. Moreover, our results are consistent with the possibility that WM connectivity alterations statistically mediate the association between leptin and NF‐L, though temporal and causal relationships cannot be inferred from cross‐sectional data. Speculatively, hypoleptinemia may contribute to neuronal damage processes in AN. A study in genetically leptin‐deficient adults has shown that exogenous leptin administration might have positive effects on GM development (Matochik et al., 2005), and the relationships seen in the present study suggest that there might also be a potential for positive effects on WM. Encouraging findings from case studies have paved the way for exploring the potential of exogenous leptin in the treatment of AN (Hebebrand et al., 2021). Future research should explore these therapeutic possibilities since they might hold the potential of mitigating the drastic effects of undernutrition on brain structure in AN.

## Ethical considerations

The study was approved by the ethics committee of the Technische Universität Dresden (protocol numbers EK 536122015, 03.01.2016/EK 39022012, 02.14.2012) and all participants (and the legal guardians of underage participants) gave written informed consent.

**Key points**

**What's known?**

- Anorexia nervosa (AN) is an eating disorder which usually has its first onset in adolescence.
- AN is associated with drastic alterations in brain structure.
- Neurofilament light (NF‐L) is a blood marker for axonal damage and has been previously shown to be related to gray matter alterations in AN.

**What's new?**

- In the present study, alterations in white matter (WM) volume as well as WM connectivity were associated with elevated blood concentrations of NF‐L.

**What's relevant?**

- Leptin was negatively correlated with NF‐L concentrations. Mediation analyses suggest that the correlation might be partially mediated by changes in WM microstructure.

## Supporting information


**Appendix S1.** Materials and methods.
**Table S1.** Parcellations used for the White Matter Volume Analysis.
**Appendix S2.** Results.
**Table S2.** Mean Distance Expressed in Standard Deviations from the Group Mean in Connectivity Values and White Matter Volume Measures of the Participants on Antidepressant Medication and of the Binge‐Purge Subtype.
**Table S3.** Correlations of Clinical Variables with NF‐L.
**Figure S1a.** Influence of Different Thresholds on the Size of the Largest Component, the Number of Significant 4 Components and the Total Amount of Significant Edges in the Fractional Anisotropy (FA) Model.
**Figure S1b.** Influence of Different Thresholds on the Size of the Largest Component, the Number of Significant 8 Components and the Total Amount of Significant Edges in the Number of Streamlines (NOS) Model.
**Table S4.** Associations of NF‐L levels with White Matter Volume.
**Figure S2.** Associations of NF‐L levels with White Matter Volume.
**Table S5.** t‐ and *p*‐values of Group Comparisons of White Matter Volume.
**Figure S3.** Network‐Based Statistic (NBS) Components where NF‐L was Associated with NOS.
**Table S6.** Associations between Mean Connectivity in the Components and Leptin Controlling for BMI‐SDS.
**Table S7.** Complete Table of Associations between Mean Connectivity in the Components and Clinical Variables excluding imputed leptin values.

## Data Availability

The data used in this study consist of sensitive patient information, and due to ethical and data privacy regulations, they cannot be shared publicly.
