# Supplementary material for: Elevated neurofilament light levels in acute anorexia nervosa are associated with alterations in white matter volume and connectivity networks
Source: J Child Psychol Psychiatry. 2025 Dec 2;67(6):907–18. doi: 10.1111/jcpp.70083 (PMC13170629; doi:10.1111/jcpp.70083)
Supplement: Supplementary file 1 — Appendix S1. Materials and methods. Table S1. Parcellations used for the White Matter Volume Analysis. Appendix S2. Results. Table S2. Mean Distance Expressed in Standard Deviations from the Group Mean in Connectivity Values and White Matter Volume Measures of the Participants on Antidepressant Medication and of the Binge‐Purge Subtype. Table S3. Correlations of Clinical Variables with NF‐L. Figure S1a. Influence of Different Thresholds on the Size of the Largest Component, the Number of Significant 4 Components and the Total Amount of Significant Edges in the Fractional Anisotropy (FA) Model. Figure S1b. Influence of Different Thresholds on the Size of the Largest Component, the Number of Significant 8 Components and the Total Amount of Significant Edges in the Number of Streamlines (NOS) Model. Table S4. Associations of NF‐L levels with White Matter Volume. Figure S2. Associations of NF‐L levels with White Matter Volume. Table S5. t‐ and p‐values of Group Comparisons of White Matter Volume. Figure S3. Network‐Based Statistic (NBS) Components where NF‐L was Associated with NOS. Table S6. Associations between Mean Connectivity in the Components and Leptin Controlling for BMI‐SDS. Table S7. Complete Table of Associations between Mean Connectivity in the Components and Clinical Variables excluding imputed leptin values. [file JCPP-67-907-s001.docx]

**Elevated Neurofilament Light Levels in acute Anorexia Nervosa are Associated with Alterations in White Matter Volume and Connectivity Networks**

**Supporting Information**

# Appendix S1. Materials and Methods

*1.1 Participants*

Normal weight in healthy control participants (HC) was defined by a body mass index (BMI) between

18.5 kg/m^2^ and 28 kg/m^2^ (or between the 10th and the 94th age percentile, in participants younger than

19 years). Additional exclusion criteria for HC were a lifetime BMI below 17.5 kg/m^2^ (or below the 10th age percentile when younger than 19 years) and substantial weight loss in the four weeks preceding study participation. Patients with anorexia nervosa (AN) were included if no substantial weight gain was reported in the four weeks preceding the first study point. In addition to the exclusion criteria mentioned in the main manuscript, participants in both groups were excluded if they had any history of organic brain syndrome, dementia, schizophrenia, psychosis, or bipolar disorder. Further exclusion criteria for all participants were an IQ below 85, current substance abuse, current inflammatory, neurologic, or metabolic illness, clinically relevant anemia, pregnancy, and breast feeding. Information relevant to inclusion/exclusion criteria, including lifetime neurological disorders and possible confounding variables was obtained using the SIAB-EX^1^, our own semi-structured interview, and medical records. Information about comorbid diagnoses in AN participants was obtained from medical records and confirmed by an expert clinician. The study sample has nearly complete overlap with the previous NBS study from our group^2^. Also other study samples from our group overlap substantially with the current sample (e.g.^3–7^).

The study was designed as a follow-up study to the study of Geisler et. al. ^2^ to further investigate the mechanisms underlying the results. We thus used the sample of Geisler et. al. but restricted it to those participants for whom we had a neurofilament light (NF-L) measurement. The sampling procedure of Geisler et. al. included an age-matching procedure using the Munkres algorithm^8^.

*1.2 Clinical Measures*

IQ was estimated using full or short versions of age-appropriate German versions of the Wechsler Intelligence Scales for adults or children (HAWIK-IV^9^ in n = 80; WISC-IV^10^ in n = 2; WIE^11^ in n = 58; WAIS-IV^12^ in n = 4, other in n = 2). Short versions were used in 89 participants. In eight cases, IQ was missing. Missing clinical measures were not imputed.

*1.3 Blood Sampling and Analysis*

Venous blood samples were collected into vacutainer tubes between 7 and 9 a.m. after an overnight fast. To yield blood serum, blood samples were left to clot for 30 min at 6–8°C and then centrifuged (2500 x g for 15 min) in a pre-cooled (5°C) centrifuge. The samples were then aliquoted into pre-cooled Eppendorf Tubes® and stored at -80°C. Determination of NF-L levels was carried out using the digital Simoa^TM^ Human Neurology 4-Plex A assay in combination with the Simoa^TM^ HD-1 Analyzer or the digital Simoa® NF-light™ Advantage Kit in combination with the Simoa^TM^ HD-X Analyzer (all

Quanterix, Lexington, MA, USA) following the manufacturer’s instructions. The Simoa^TM^ technology reaches significantly lower limits of detection compared to conventional assays^13,14^. Limit of detection for NF-L was 0.104 pg/ml using the Human Neurology 4-Plex A assay and 0.038 pg/ml using the NFlight^TM^ Advantage Kit^15,16^. The samples were measured in different batches and since the coefficient of variation in the first batches measured in duplicates (n = 107) was always very good (see previous studies: Hellerhoff et. al. 2021^4^, Doose et. al. 2021^17^, Hellerhoff et. al. 2023^5^), the later samples were measured in singlicates (n = 48).

For blood plasma processing, aprotinin was added during blood sampling (270 KIU/ml final concentration). Plasma samples were centrifuged immediately (2500 x g for 15 min) in a pre-cooled

(5°C) centrifuge. Thereafter they were aliquoted into pre-cooled Eppendorf Tubes® and stored at -80°C. Plasma leptin was measured in singlicates using a commercially available enzyme-linked immunosorbent assay (BioVendor, Brno, Czech Republic). Left-censored leptin concentrations below the lower limit of detection of the applied leptin assay (LOD = 0.20 ng/mL; n = 17 AN and 0 HC) were imputed using a quantile regression multiple imputation approach for left-censored missing data (QRILC). QRILC performs random draws from a truncated distribution with parameters estimated using quantile regression (derived from the distribution of existing leptin concentrations within detection range, please note that leptin values were log10-transformed for the imputation procedure and that no further covariates were introduced in the imputation model). QRILC was conducted in R with the help of package “imputeLCMD”^18^. A Gibbs sampler based approach^19^ with n = 100 iterative draws per value from the specified truncated distribution was then used to update the initialized values from QRILC and to ensure that the imputed leptin values were positive (on the original scale, i.e., > 0) and below LOD.

*1.4 MRI Acquisition and Processing*

1.4.1 MRI acquisition

As in our previous studies^3,20^, high-resolution three-dimensional T1-weighted structural scans were acquired on a 3T scanner (Magnetom Trio, Siemens, Erlangen, Germany) using a rapid acquisition gradient echo (MP-RAGE) sequence with the following parameters: 176 sagittal slices (1 mm thickness, no gap), TR = 1900 ms; TE = 2.26 ms; flip angle = 9°; voxel size = 1.0 × 1.0 × 1.0 mm^3^, FoV = 256 × 224 mm^2^, bandwidth of 200 Hz/pixel).

Diffusion-weighted imaging data were collected using a spin-echo sequence at 2.4 mm isotropic voxel resolution, 307x307x144 mm3 FoV, 128x128 matrix size, 60 slices, no inter-slice gap, TE = 104 milliseconds, TR = 15 seconds, BW = 2056 Hz/Px, GRAPPA acceleration factor 2, 24 reference lines, and prescan normalize. A total of 32 diffusion sensitizing gradients (b = 1,300 s/mm2) were applied, and 4 images without diffusion weighting (b = 0 s/mm2) were acquired.

1.4.2 MRI Preprocessing and Quality Control (QC)

1.4.2.1 WM volume Analyses

MRI preprocessing and quality control (QC) for the WM volume analyses were performed using standard FreeSurfer (version 5.3) procedures to extract measures of volumes of WM in regions of interest (ROI)^21–26^. Surface reconstruction was first performed for each hemisphere. For the subcortical volumetric analyses, we used the automated segmentation procedures implemented in the FreeSurfer pipeline to assign an anatomical label to each voxel based on probabilistic information estimated from a manually labeled training set (https://surfer.nmr.mgh.harvard.edu/fswiki/asegstats2table). The quality of the segmentation was assured by visual inspection by a trained examiner with the support of quality assurance tools implemented in FreeSurfer ([https://surfer.nmr.mgh.harvard.edu/fswiki/QATools)](https://surfer.nmr.mgh.harvard.edu/fswiki/QATools) and participants who did not meet quality criteria were excluded a priori from the analyses. Table SI 1

displays the parcellations analyzed in the WM volume analysis.

1. **Table S1**
2. *Parcellations used for the White Matter Volume Analysis*

| **Left hemisphere** lhCorticalWhiteMatterVol wm.lh.bankssts wm.lh.caudalanteriorcingulate wm.lh.caudalmiddlefrontal wm.lh.cuneus wm.lh.entorhinal wm.lh.fusiform wm.lh.inferiorparietal wm.lh.inferiortemporal wm.lh.isthmuscingulate wm.lh.lateraloccipital wm.lh.lateralorbitofrontal wm.lh.lingual wm.lh.medialorbitofrontal wm.lh.middletemporal wm.lh.parahippocampal wm.lh.paracentral wm.lh.parsopercularis wm.lh.parsorbitalis wm.lh.parstriangularis wm.lh.pericalcarine wm.lh.postcentral wm.lh.posteriorcingulate wm.lh.precentral wm.lh.precuneus wm.lh.rostralanteriorcingulate wm.lh.rostralmiddlefrontal wm.lh.superiorfrontal wm.lh.superiorparietal wm.lh.superiortemporal wm.lh.supramarginal wm.lh.frontalpole wm.lh.temporalpole wm.lh.transversetemporal wm.lh.insula  Left.UnsegmentedWhiteMatter | **Right hemisphere** rhCorticalWhiteMatterVol wm.rh.bankssts wm.rh.caudalanteriorcingulate wm.rh.caudalmiddlefrontal wm.rh.cuneus wm.rh.entorhinal wm.rh.fusiform wm.rh.inferiorparietal wm.rh.inferiortemporal wm.rh.isthmuscingulate wm.rh.lateraloccipital wm.rh.lateralorbitofrontal wm.rh.lingual wm.rh.medialorbitofrontal wm.rh.middletemporal wm.rh.parahippocampal wm.rh.paracentral wm.rh.parsopercularis wm.rh.parsorbitalis wm.rh.parstriangularis wm.rh.pericalcarine wm.rh.postcentral wm.rh.posteriorcingulate wm.rh.precentral wm.rh.precuneus wm.rh.rostralanteriorcingulate wm.rh.rostralmiddlefrontal wm.rh.superiorfrontal wm.rh.superiorparietal wm.rh.superiortemporal wm.rh.supramarginal wm.rh.frontalpole wm.rh.temporalpole wm.rh.transversetemporal wm.rh.insula  Right.UnsegmentedWhiteMatter | **Total brain**  CorticalWhiteMatterVol  (MaskVol)^a^  (EstimatedTotalIntraCranialVol)^a^ |
| --- | --- | --- |

1. *Note.*^a^The parcellations “MaskVol” and “EstimatedTotalIntracranialVol” were not included in the analysis since not adherent 4 to the research question.

5

1. 1.4.2.2 Preprocessing for NBS Analyses
2. For the diffusion weighted imaging (DWI) analyses, after converting to the Nifti format, the raw data
3. were analyzed in an automated manner with the standard FreeSurfer pipeline
4. (http://surfer.nmr.mgh.harvard.edu, version 5.1.0). This automatic process (recon-all) included the

preprocessing of the data (e.g. intensity normalization, skull stripping) and also the volumetric segmentation as well as the cortical surface reconstruction. Surface reconstruction was performed for each hemisphere and included tessellation of the gray matter-white matter boundary, automated topology correction, and surface deformation following intensity gradients to optimally place the graywhite and gray-cerebrospinal fluid borders at the location where the greatest shift in intensity defines the transition to the other tissue class. After the successful run of recon-all, the quality of the surface reconstruction and segmentation was assured according to FreeSurfer user guidelines by visual inspection.

Then, we used a high-resolution subdivision of the Desikan-Killiany atlas (also known as Lausanne2008 atlas)^21,22^ to assign each cortical surface vertex a neuroanatomical label. This atlas is available at five different scales of equally sized ROI. In our study, we chose the medium scale of the Lausanne2008 containing 233 grey matter regions (219 cortical and 14 subcortical). The Lausanne2008 atlas is included in the Connectome Mapper ToolKit^23^ and is available on [https://github.com/mattcieslak/easy_lausanne.](https://github.com/mattcieslak/easy_lausanne)

We used the following variables from the standard output of FreeSurfer: CorticalWhiteMatterVol (total volume of cortical WM), and EstimatedTotalIntraCranialVol (intracranial volume).

The DWI pre-processing pipeline has been described in detail previously^24^ and involved the following steps: (1) diffusion-weighted data were realigned, corrected for Eddy current-induced distortions and participant movements using Eddy from FSL (https://fsl.fmrib.ox.ac.uk). The output of the estimated frame-wise motion parameter was averaged across volumes and later used as nuisance variable. (2) We fitted a diffusion profile within each voxel using 32 weighted images and the average *b* = 0 image from each participant.

From the resulting tensor, the main diffusion direction in each voxel was computed as the principal eigenvector resulting from the eigenvalue decomposition of the fitted tensor, marking the preferred diffusion direction in each voxel. For each voxel, the fractional anisotropy (FA) was computed.

The fiber tracking and network construction procedures are described in the main manuscript (2.4.2.2. and 2.4.2.3).

For outlier detection in the DWI data, we used Carling’s criterion^25^. The rigorous data-driven QC on connectivity matrices was carried out to minimize the chance of non-anatomical connections. First, we classified edges as missing (on the subject level) if there were less than three fibers touching the two associated regions.

For outlier detection, we calculated the average connectivity by averaging the connectivity matrices, resulting in mean values for number of streamlines (NOS) and FA for each participant. The prevalence of an edge is the percentage of this edge existing in its reference group (AN or HC of the bigger sample used in Geisler et al.^2^). By applying the Carling criterion for outlier detection, a participant was marked as an outlier and excluded if (1) a participant’s average connectivity value was an outlier in the reference group, or (2) the average frame-wise motion (taken from Eddy output) was an outlier in its reference group, or (3) the prevalence of existing or non-existent edges was an outlier in its reference group. Then the final connectivity matrices were thresholded such that only edges with a prevalence of > 60% across all participants were included^26^. This procedure minimizes the chance of spurious connections, based on the assumption that connections consistently detected in a large percentage of the subjects are more likely to exist than connections detected in only a few subjects. The applied prevalence threshold of 60% provides a good balance between the elimination of false positives and false negatives^27^.

For the NBS procedures the motion covariate was calculated as the average frame-wise motion estimates from EDDY correction across volumes.

*1.5 Statistical Analyses*

1.5.1 Group Comparisons of Demographic Variables

For the analysis of demographic variables, non-parametric tests (Wilcoxon rank sum tests) were used since not all variables were normally distributed. Group comparisons of log-transformed NF-L levels were conducted using Welch two sample t-tests (with correction of degrees of freedom to account for differences between groups in the variance). Kendall’s tau was computed to test for correlations between demographic variables and a follow-up robust linear model (rlm from the MASS package followed by f.robtest from the sfsmisc package for follow-up tests) was used to test whether the association between NF-L and leptin remained significant after controlling for BMI. False discovery rate (FDR)^28^ correction was applied to account for multiple testing (details in table 1 of the main manuscript).

1.5.2 Analysis of Group Differences in WM Volume Measures

Group differences in WM volume measures were explored using linear models predicting WM volume from group, age, and eTIV (the latter two as control variables). FDR correction was applied to account for multiple comparisons (correction over 73 tests).

1.5.3 Relationship to Clinical Variables (WM Volume and NBS)

To test associations with clinical variables, robust linear models were fitted using an *M*-estimator^29^, which is effective in dealing with various violations of the assumptions of the classical linear model. The robust linear models were fitted using the MASS package for R^30,31^.

1.5.4 Sensitivity Analyses Investigating the Potential Influence of Antidepressant Medication and Subtype

To assure that the results were not driven by participants on antidepressant medication (n = 3) or of the binge-purge subtype (n = 8), we performed additional analyses. For each of those participants, we related the connectivity values in each component and the WM volume values for the whole brain and the left and right hemispheres, respectively, to the mean and standard deviation of the complete sample to see whether these participants might have driven the results.

1.5.5 Sensitivity Analyses Investigating the Potential Influence of imputed leptin values

To test, whether the analyses of relationship between illness-related variables and NBS components in AN were influenced by the imputed leptin values, a secondary analysis excluding all imputed leptin was conducted (table S7).

1.5.6 Software

Analysis of demographic and clinical variables and of damage marker levels as well as the analyses of WM volume and of relationships between connectivity networks/WM volume and clinical variables was carried out using the software R (version 4.3.1)^31^. The mediation analysis was performed using the R

package “Robmed”^32^. NBS was carried out using Networkx^33^.

# Appendix S2. Results

*2.1 Sensitivity Analyses Investigating the Potential Influence of Antidepressant Medication and Subtype* To assure that the results were not driven by participants on antidepressant medication (n = 3) or of the binge-purge subtype (n = 8), we performed additional analyses. For each of those participants, we investigated whether they might have influenced the results by inspecting if the connectivity values in each component and the WM volume values for the whole brain and the left and right hemispheres, were within 3 SD of the sample mean.

For none of the participants on antidepressant medication or of the binge-purge subtype, the connectivity values in any component or the WM volume values for the whole brain and the left and right hemispheres were to be considered as outliers (distance to the mean of 3 standard deviations or more).

**Table S2**

*Mean Distance Expressed in Standard Deviations from the Group Mean in Connectivity Values and White Matter Volume Measures of the Participants on Antidepressant Medication and of the Binge-Purge Subtype*

|  | **Participants on antidepressant medication** | **Participants of the binge-purge subtype** |
| --- | --- | --- |
| **Connectivity component CFA1** | -0.322 | 0.259 |
| **Connectivity component CFA2** | -0.071 | -0.339 |
| **Connectivity component CFA3** | -0.347 | 0.203 |
| **Connectivity component CFA4** | 0.105 | 0.429 |
| **Connectivity component CFA5** | 0.418 | 0.008 |
| **Connectivity component CFA6** | 0.316 | -0.246 |
| **Connectivity component CFA7** | -0.441 | 0.155 |
| **Cortical WM volume** | -0.364 | 0.259 |
| **Cortical WM volume lh** | -0.470 | -0.339 |
| **Cortical WM volume rh** | -0.259 | 0.203 |

1. *Note.* lh = left hemisphere; rh = right hemisphere.; WM = white matter
2. *2.2 Correlations of Clinical Variables with NF-L*
3. **Table S3**
4. *Correlations of Clinical Variables with NF-L*

| **Variable** | **AN** |  | **HC** |  |
| --- | --- | --- | --- | --- |
|  | **Kendall’s tau** | **p** | **Kendall’s tau** | **p** |
| **Age** | -0.048 | .538 | 0.010 | .902 |
| **IQ** | -0.010 | .903 | 0.047 | .570 |
| **BMI** | -0.304 | < .001*** | -0.049 | .526 |
| **BMI-SDS** | -0.233 | .003* | -0.029 | .712 |
| **Minimal lifetime**  **BMI** | -0.205 | .009* | -0.105 | .178 |
| **EDI-2 “Drive for thinness”** | 0.023 | .769 | 0.041 | .605 |
| **EDI-2 “Body**  **dissatisfaction”** | 0.059 | .456 | 0.059 | .453 |
| **BDI-II** | < 0.001 | .996 | < 0.001 | .996 |

**Leptin** -0.321 < .001*** 0.035 .660

1. *Note.* AN = participants with acute anorexia nervosa; HC = healthy control participants; BMI = body mass index; BMI-SDS = 6 body mass index standard deviation score; EDI-2 = Eating Disorder Inventory, version 2; BDI-II = Beck Depression Inventory, 7 version 2; The asterisks denote the following significance levels after false discovery rate (FDR) correction for 9 tests in each

8 group: * FDR < .05 ** FDR < .01 ***FDR < .001.

*2.3 Results of the Robust Linear Regression Predicting NF-L from Leptin and BMI-SDS*

1. The robust linear model indicated an association between NF-L and leptin even when controlling for
2. BMI-SDS (robust linear regression: *β* = -1.136, *t* = -3.120; robust F test on the robust linear regression:
3. *F* = 10.058, *p* = .002).

5

*2.4 Plots of different thresholds for the NBS models*

**Figure S1a**

3 *Influence of Different Thresholds on the Size of the Largest Component, the Number of Significant* 4 *Components and the Total Amount of Significant Edges in the Fractional Anisotropy (FA) Model*

5


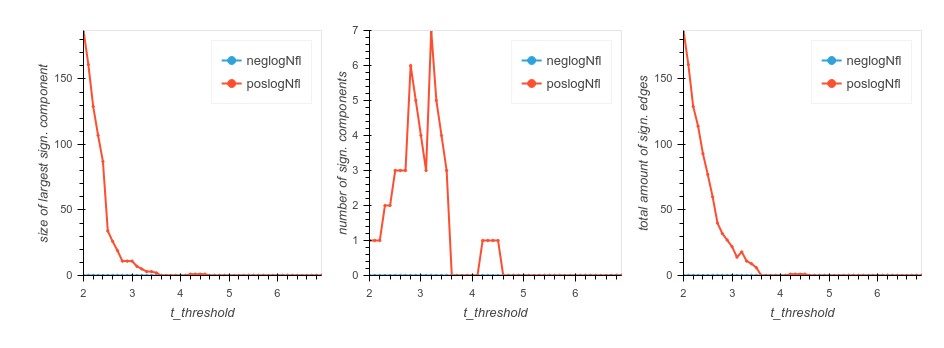


1. **Figure S1b**
2. *Influence of Different Thresholds on the Size of the Largest Component, the Number of Significant* 8 *Components and the Total Amount of Significant Edges in the Number of Streamlines (NOS) Model*

9


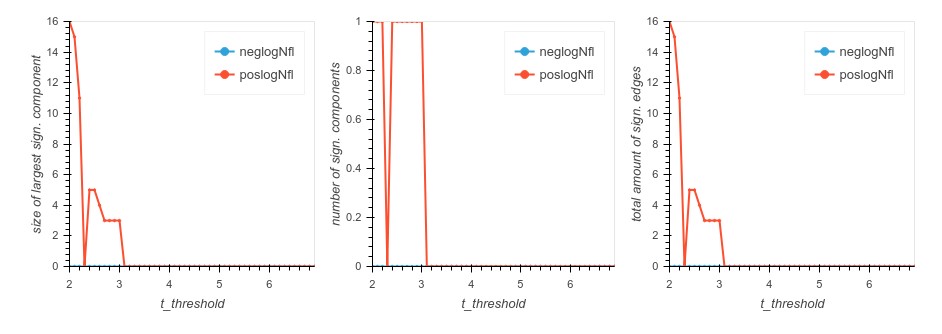


10

*2.5 Associations of NF-L Levels with White Matter Volume*

1. **Table S4**
2. *Associations of NF-L levels with White Matter Volume*

|  | **AN** |  |  | **HC** |  |
| --- | --- | --- | --- | --- | --- |
| **parcellation** | **β** | **p** | **β** |  | **p** |
| **left hemisphere** |  |  |  |  |  |
| lhCorticalWhiteMatterVol | 24338.787 | < .001** | 20009.114 |  | .041 |
| wm.lh.bankssts | -458.655 | .162 | -433.346 |  | .343 |
| wm.lh.caudalanteriorcingulate | -179.043 | .315 | 391.371 |  | .174 |
| wm.lh.caudalmiddlefrontal | -724.242 | .075 | 1313.328 |  | .039 |
| wm.lh.cuneus | -164.449 | .407 | 1012.326 |  | < .001* |
| wm.lh.entorhinal | -53.450 | .559 | 306.463 |  | .042 |
| wm.lh.fusiform | -851.282 | .005* | -185.288 |  | .706 |
| wm.lh.inferiorparietal | -340.155 | .568 | 1658.308 |  | .042 |
| wm.lh.inferiortemporal | -817.865 | .031 | 967.515 |  | .076 |
| wm.lh.isthmuscingulate | -273.239 | .173 | 1014.914 |  | .002 |
| wm.lh.lateraloccipital | -925.736 | .086 | 81.362 |  | .907 |
| wm.lh.lateralorbitofrontal | -261.843 | .314 | 666.218 |  | .099 |
| wm.lh.lingual | -331.344 | .311 | 780.689 |  | .148 |
| wm.lh.medialorbitofrontal | -490.281 | .077 | 707.593 |  | .139 |
| wm.lh.middletemporal | -335.542 | .306 | 687.345 |  | .144 |
| wm.lh.parahippocampal | -268.271 | .009 | 95.823 |  | .542 |
| wm.lh.paracentral | -661.972 | .005* | 119.555 |  | .713 |
| wm.lh.parsopercularis | -436.766 | .055 | 701.416 |  | .044 |
| wm.lh.parsorbitalis | -110.604 | .069 | -10.898 |  | .907 |

wm.lh.parstriangularis -468.557 .037 488.947 .082

| wm.lh.pericalcarine | -304.935 | .332 | 513.096 | .218 |
| --- | --- | --- | --- | --- |
| wm.lh.postcentral | -847.693 | .016 | -794.956 | .133 |
| wm.lh.posteriorcingulate | -131.587 | .502 | 7.327 | .982 |
| wm.lh.precentral | -1989.726 | .003* | 1269.966 | .109 |
| wm.lh.precuneus | -955.052 | .058 | 1154.794 | .077 |
| wm.lh.rostralanteriorcingulate | -143.148 | .428 | 26.202 | .925 |
| wm.lh.rostralmiddlefrontal | -867.198 | .125 | 617.802 | .432 |
| wm.lh.superiorfrontal | -2285.745 | .002* | 1300.336 | .245 |
| wm.lh.superiorparietal | -1331.603 | .031 | 816.154 | .375 |
| wm.lh.superiortemporal | -293.554 | .402 | 1264.533 | .077 |
| wm.lh.supramarginal | 115.798 | .828 | -159.185 | .838 |
| wm.lh.frontalpole | -46.334 | .076 | -27.237 | .500 |
| wm.lh.temporalpole | -48.984 | .380 | 99.842 | .311 |
| wm.lh.transversetemporal | -111.474 | .115 | -62.805 | .578 |
| wm.lh.insula | -682.583 | .017 | 500.132 | .321 |
| Left.UnsegmentedWhiteMatter | -4853.949 | .013 | 3348.141 | .351 |
| **right hemisphere** |  |  |  |  |
| rhCorticalWhiteMatterVol | -23306.922 | < .001** | 20529.462 | .035 |
| wm.rh.bankssts | -270.614 | .299 | -248.942 | .491 |
| wm.rh.caudalanteriorcingulate | -279.525 | .087 | -222.793 | .460 |
| wm.rh.caudalmiddlefrontal | -1328.280 | .002* | 671.888 | .244 |
| wm.rh.cuneus | -161.372 | .414 | 568.838 | .043 |
| wm.rh.entorhinal | 22.111 | .775 | -88.018 | .995 |
| wm.rh.fusiform | -1052.605 | < .001* | -177.839 | .719 |
| wm.rh.inferiorparietal | -617.897 | .346 | 962.744 | .282 |
| wm.rh.inferiortemporal | -930.195 | .007* | -156.425 | .706 |

wm.rh.isthmuscingulate 94.390 .527 291.285 .306

| wm.rh.lateraloccipital | -157.092 | .759 | 1234.885 | .089 |
| --- | --- | --- | --- | --- |
| wm.rh.lateralorbitofrontal | -391.850 | .119 | 861.815 | .070 |
| wm.rh.lingual | -452.939 | .212 | 699.504 | .189 |
| wm.rh.medialorbitofrontal | -17.841 | .923 | 106.105 | .713 |
| wm.rh.middletemporal | -397.300 | .168 | 698.661 | .127 |
| wm.rh.parahippocampal | -123.632 | .330 | 197.447 | .202 |
| wm.rh.paracentral | -555.071 | .060 | -19.560 | .965 |
| wm.rh.parsopercularis | -431.066 | .086 | 136.545 | .724 |
| wm.rh.parsorbitalis | -110.079 | .142 | -63.416 | .582 |
| wm.rh.parstriangularis | -572.762 | .036 | 134.749 | .731 |
| wm.rh.pericalcarine | 85.835 | .808 | 636.279 | .088 |
| wm.rh.postcentral | -935.194 | .013 | 70.130 | .885 |
| wm.rh.posteriorcingulate | -403.510 | .045 | 526.192 | .102 |
| wm.rh.precentral | -1437.063 | .022 | 1217.215 | .248 |
| wm.rh.precuneus | -1382.181 | .008* | 1320.467 | .123 |
| wm.rh.rostralanteriorcingulate | -231.897 | .062 | 1.965 | .992 |
| wm.rh.rostralmiddlefrontal | -401.359 | .517 | 2351.608 | .004 |
| wm.rh.superiorfrontal | -1792.241 | .006* | 1560.577 | .175 |
| wm.rh.superiorparietal | -888.044 | .118 | 235.909 | .815 |
| wm.rh.superiortemporal | -350.529 | .213 | 685.395 | .234 |
| wm.rh.supramarginal | -806.674 | .095 | 196.038 | .798 |
| wm.rh.frontalpole | -1.824 | .962 | 43.799 | .356 |
| wm.rh.temporalpole | -24.191 | .660 | 26.846 | .790 |
| wm.rh.transversetemporal | -74.122 | .141 | 131.072 | .041 |
| wm.rh.insula | -719.756 | .082 | 757.249 | .139 |
| Right.UnsegmentedWhiteMatter | -4268.745 | .031 | 3534.931 | .328 |

**Global**

CorticalWhiteMatterVol

-

47645.709

< .001**

40538.576

.036

*Note.* The table shows the estimates (β) and p-values for neurofilament light (NF-L) as a predictor in the regressions predicting the denoted WM volume measures (controlling for age and estimated total intracranial volume). WM volume is indicated in mm^3^.The asterisks denote the following significance levels after false discovery rate (FDR) correction for 73 tests: * FDR < .05 ** FDR < .01.

1. **Figure S2**
2. *Associations of NF-L levels with White Matter Volume*
3.
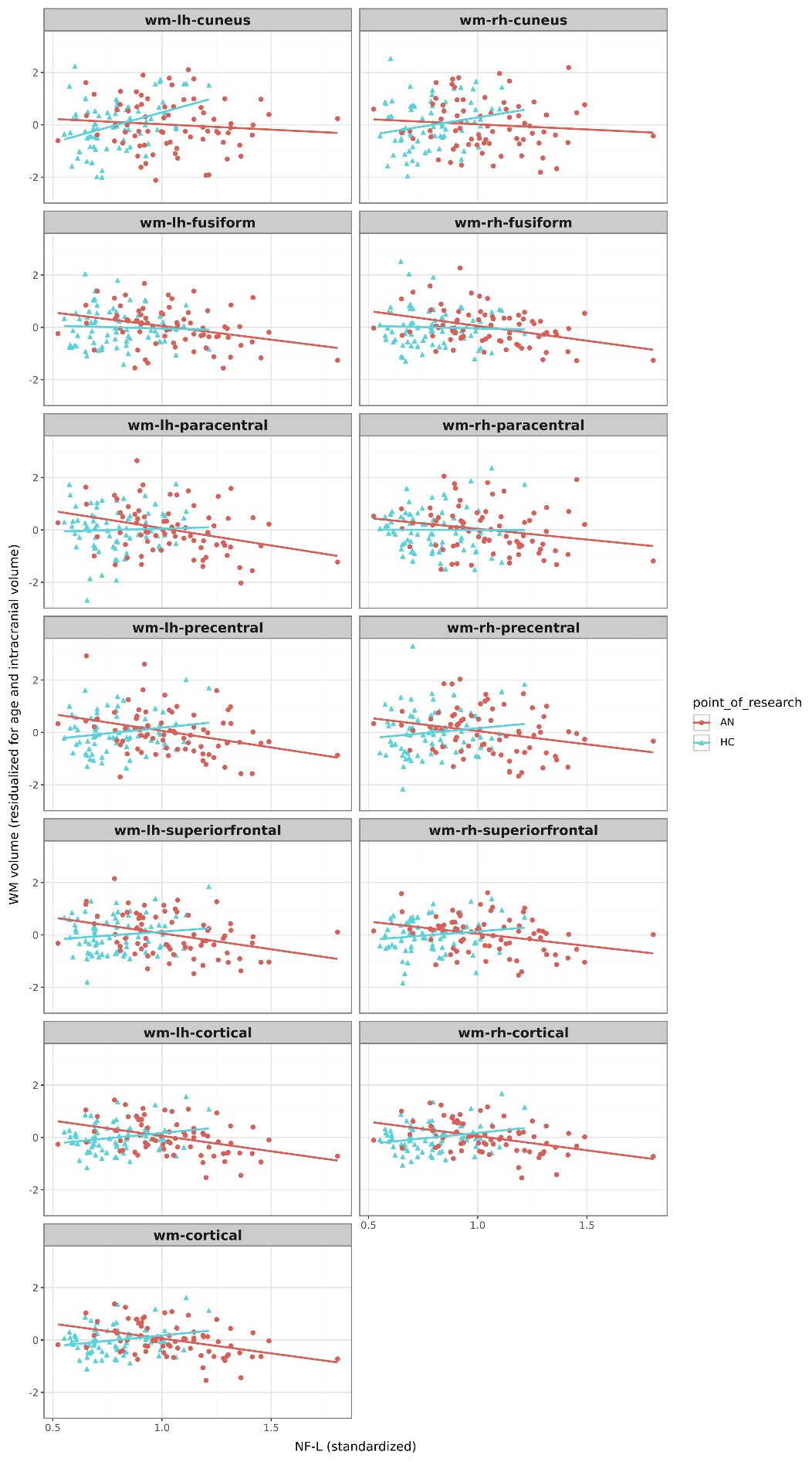
 1
4. *2.6 Supplementary Analyses of Group Differences in White Matter Volume*
5. **Table S5**
6. *t- and p-values of Group Comparisons of White Matter Volume*

|  | **left hemisphere** | | | **right hemisphere** | |
| --- | --- | --- | --- | --- | --- |
| **parcellation** | **t** | **p** | **t** | | **p** |
| CorticalWhiteMatterVol | 0.821 | .413 | 1.076 | | .284 |
| bankssts | 0.437 | .663 | 0.726 | | .469 |
| caudalanteriorcingulate | 0.266 | .791 | 1.056 | | .293 |
| caudalmiddlefrontal | 0.560 | .576 | -0.849 | | .397 |
| cuneus | -1.223 | .223 | -0.443 | | .658 |
| entorhinal | 1.312 | .192 | 0.644 | | .520 |
| fusiform | 0.819 | .414 | -0.853 | | .395 |
| inferiorparietal | 1.782 | .077 | 1.223 | | .223 |
| inferiortemporal | 0.861 | .391 | 0.743 | | .459 |
| isthmuscingulate | -0.243 | .808 | -1.311 | | .192 |
| lateraloccipital | -1.824 | .070 | -1.404 | | .162 |
| lateralorbitofrontal | 1.211 | .228 | 2.001 | | .047 |
| lingual | -0.031 | .976 | 0.599 | | .550 |
| medialorbitofrontal | -0.535 | .593 | 0.407 | | .685 |
| middletemporal | 1.019 | .310 | 1.548 | | .124 |
| parahippocampal | 1.936 | .055 | 0.076 | | .939 |
| paracentral | 1.992 | .048 | 2.029 | | .044 |
| parsopercularis | 0.117 | .907 | -0.874 | | .384 |
| parsorbitalis | 0.515 | .607 | 1.699 | | .092 |
| parstriangularis | -0.484 | .629 | 1.171 | | .244 |

pericalcarine 0.454 .651 -0.274 .784

| postcentral | 0.942 |  | .348 | -0.635 |  | .526 |
| --- | --- | --- | --- | --- | --- | --- |
| posteriorcingulate | 0.801 |  | .425 | 1.531 |  | .128 |
| precentral | 1.153 |  | .251 | 1.686 |  | .094 |
| precuneus | 0.692 |  | .490 | -0.465 |  | .642 |
| rostralanteriorcingulate | -0.114 |  | .910 | 1.987 |  | .049 |
| rostralmiddlefrontal | -0.441 |  | .660 | 1.500 |  | .136 |
| superiorfrontal | 0.662 |  | .509 | 0.949 |  | .344 |
| superiorparietal | 0.073 |  | .942 | 0.655 |  | .513 |
| superiortemporal | -0.771 |  | .442 | -0.346 |  | .730 |
| supramarginal | -1.955 |  | .053 | -1.923 |  | .056 |
| frontalpole | 0.962 |  | .338 | -0.640 |  | .523 |
| temporalpole | 2.630 |  | .009 | 1.120 |  | .264 |
| transversetemporal | 1.025 |  | .307 | -0.621 |  | .535 |
| insula | 0.668 |  | .505 | -0.451 |  | .653 |
| UnsegmentedWhiteMatter | 0.497 |  | .620 | 1.052 |  | .294 |
| **Global** |  | **t** |  |  | **p** |  |

CorticalWhiteMatterVol 0.954 .342

1. *Note.* The table shows the t- and p-values for group comparisons of white matter volume (controlling for age and estimated
2. total intracranial volume).
   1. *Association of NF-L with Connectivity Networks in HC*

**Figure S3**

*Network-Based Statistic (NBS) Components where NF-L was Associated with NOS*


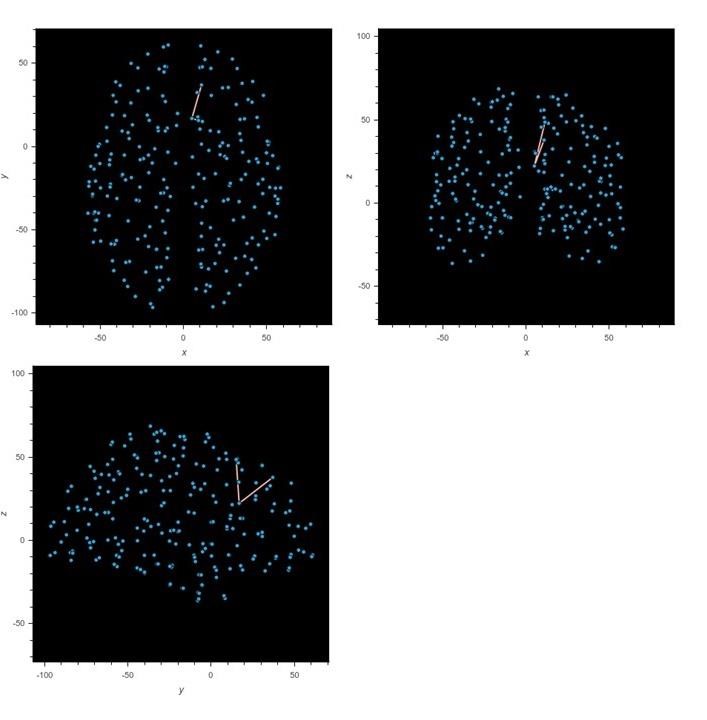


*Note.* Results of the NBS at the primary threshold of t = 3.2 are shown. The component in which neurofilament light (NF-L) was positively associated with logarithmized number of streamlines (NOS) in healthy control (HC) participants is depicted as connected edges of the same color.

- 1. *Associations between Illness-related Variables and NBS Components in AN*

To control for potential overlapping effects between the association of mean connectivity with leptin an

1. BMI-SDS, an additional analysis was performed, including BMI-SDS as a regressor (results in table
2. ST5).
3. **Table S6**
4. *Associations between Mean Connectivity in the Components and Leptin Controlling for BMI-SDS*

| **Predictor** | **Component** | **β** | **p** |
| --- | --- | --- | --- |
| Leptin | CFA1 | -0.389 | 0.005* |
| Leptin | CFA2 | -0.322 | 0.015* |
| Leptin | CFA3 | -0.287 | 0.029* |
| Leptin | CFA5 | -0.304 | < .001** |

Leptin CFA6 -0.341 .005*

1. *Note.* The table shows the estimates (β) and p-values for leptin as a predictor in the robust regression models (controlling for
2. BMI-SDS) predicting mean connectivity in components with a significant association with neurofilament light (NF-L) in 9 anorexia nervosa. The asterisks denote significance levels after false discovery rate (FDR) correction (7 tests per variable): *

10 FDR < .05 ** FDR < .01.

*2.9 Sensitivity Analyses Investigating the Potential Influence of imputed leptin values*

1. **Table S7**
2. *Complete Table of Associations between Mean Connectivity in the Components and Clinical Variables*
3. *excluding imputed leptin values*

| **Predictor** | **Component** | **β** | **p** |
| --- | --- | --- | --- |
| BMI-SDS | CFA1 | -0.115 | .441 |
| BMI-SDS | CFA2 | -0.165 | .215 |
| BMI-SDS | CFA3 | -0.321 | .029 |
| BMI-SDS | CFA4 | -0.082 | .561 |
| BMI-SDS | CFA5 | -0.022 | .763 |
| BMI-SDS | CFA6 | -0.243 | .044 |
| BMI-SDS | CFA7 | -0.140 | .340 |
| Duration of illness | CFA1 | 0.084 | .587 |
| Duration of illness | CFA2 | 0.139 | .308 |
| Duration of illness | CFA3 | 0.079 | .614 |
| Duration of illness | CFA4 | 0.113 | .391 |
| Duration of illness | CFA5 | -0.092 | .217 |
| Duration of illness | CFA6 | 0.054 | .672 |
| Duration of illness | CFA7 | -0.072 | .632 |
| Leptin | CFA1 | -0.391 | .005* |
| Leptin | CFA2 | -0.354 | .008* |
| Leptin | CFA3 | -0.317 | .012* |
| Leptin | CFA4 | -0.105 | .392 |
| Leptin | CFA5 | -0.256 | .001** |
| Leptin | CFA6 | -0.351 | .003** |
| Leptin | CFA7 | -0.246 | .082 |
| BDI-II | CFA1 | -0.014 | .909 |

BDI-II CFA2 -0.165 .177

| BDI-II | CFA3 | -0.122 | .328 |
| --- | --- | --- | --- |
| BDI-II | CFA4 | 0.116 | .325 |
| BDI-II | CFA5 | 0.0468 | .490 |
| BDI-II | CFA6 | -0.138 | .203 |
| BDI-II | CFA7 | 0.041 | .747 |
| EDI-2 “Drive for thinness” | CFA1 | 0.102 | .438 |
| EDI-2 “Drive for thinness” | CFA2 | -0.063 | .604 |
| EDI-2 “Drive for thinness” | CFA3 | -0.056 | .659 |
| EDI-2 “Drive for thinness” | CFA4 | 0.127 | .300 |
| EDI-2 “Drive for thinness” | CFA5 | 0.033 | .636 |
| EDI-2 “Drive for thinness” | CFA6 | -0.188 | .082 |
| EDI-2 “Drive for thinness” | CFA7 | -0.082 | .541 |
| EDI-2 “Body dissatisfaction” | CFA1 | 0.091 | .474 |
| EDI-2 “Body dissatisfaction” | CFA2 | -0.097 | .410 |
| EDI-2 “Body dissatisfaction” | CFA3 | 0.056 | .647 |
| EDI-2 “Body dissatisfaction” | CFA4 | 0.185 | .120 |
| EDI-2 “Body dissatisfaction” | CFA5 | 0.013 | .842 |
| EDI-2 “Body dissatisfaction” | CFA6 | -0.146 | .182 |

EDI-2 “Body dissatisfaction” CFA7 -0.058 .659

1 *Note.* The table shows the estimates (β) and p-values for illness-related variables as predictors in the robust regression models 2 predicting mean connectivity in components with a significant association with neurofilament light (NF-L) in anorexia nervosa. 3 BMI-SDS = body mass index standard deviation score; EDI-2 = Eating Disorder Inventory, version 2; BDI-II = Beck 4 Depression Inventory, version 2. The asterisks denote significance levels after false discovery rate (FDR) correction (7 tests 5 per variable): * FDR < .05 ** FDR < .01.

6

# References

1. Fichter, M. & Quadflieg, N. *SIAB. Strukturiertes Inventar Für Anorektische Und Bulimische*

*Essstörungen Nach DSM-IV Und ICD-10*. (Huber, Bern, 1999).

1. Geisler, D. *et al.* Altered White Matter Connectivity in Young Acutely Underweight Patients With

Anorexia Nervosa. *J. Am. Acad. Child Adolesc. Psychiatry* **61**, 331–340 (2022).

1. Bahnsen, K. *et al.* Dynamic Structural Brain Changes in Anorexia Nervosa: A Replication Study, Mega-analysis, and Virtual Histology Approach. *J. Am. Acad. Child Adolesc. Psychiatry* **61**, 1168– 1181 (2022).
2. Hellerhoff, I. *et al.* Differential longitudinal changes of neuronal and glial damage markers in anorexia nervosa after partial weight restoration. *Transl. Psychiatry* **11**, (2021).
3. Hellerhoff, I. *et al.* Serum neurofilament light concentrations are associated with cortical thinning in anorexia nervosa. *Psychol. Med.* 1–9 (2023) doi:10.1017/S0033291723000387.
4. Pfuhl, G. *et al.* Preserved white matter microstructure in young patients with anorexia nervosa?:

Preserved White Matter Microstructure in AN. *Hum. Brain Mapp.* **37**, 4069–4083 (2016).

1. Von Schwanenflug, N. *et al.* Dynamic changes in white matter microstructure in anorexia nervosa: findings from a longitudinal study. *Psychol. Med.* **49**, 1555–1564 (2019).
2. Munkres, J. Algorithms for the assignment and transportation problems. *J. Soc. Ind. Appl. Math.* **5**,

32–38 (1957).

1. Petermann, F. & Petermann, U. *HAWIK-IV. Hamburg-Wechsler-Intelligenztest Für Kinder IV*. (Huber, Bern, 2007).
2. Petermann, F. & Petermann, U. *WISC-IV. Wechsler Intelligence Scale for Children - Fourth*

*Edition*. (Pearson Assessment & Information, Frankfurt, 2011).

1. von Aster, M., Neubauer, A. & Horn, R. *WIE. Wechsler Intelligenztest Für Erwachsene*. (Pearson

Assessment & Information, Frankfurt, 2008).

1. Petermann, F. *WAIS-IV. Wechsler Adult Intelligence Scale*. (Pearson Assessment & Information,

Frankfurt, 2012).

1. Wilson, D. H. *et al.* The Simoa HD-1 Analyzer: A Novel Fully Automated Digital Immunoassay

Analyzer with Single-Molecule Sensitivity and Multiplexing. *J. Lab. Autom.* **21**, 533–547 (2016).

1. Kuhle, J. *et al.* Comparison of three analytical platforms for quantification of the neurofilament light chain in blood samples: ELISA, electrochemiluminescence immunoassay and Simoa. *Clin.*

*Chem. Lab. Med.* **54**, 1655–1661 (2016).

1. Quanterix. Human Neurology 4-Plex ‘A’. NF-light®, Tau, GFAP*, UCHL-1*. (2017).
2. Quanterix. Simoa® NF-light^TM^ Advantage Kit: HD-1/HD-X Data Sheet. (2018).
3. Doose, A. *et al.* Neural and glial damage markers in women after long-term weight-recovery from anorexia nervosa. *Psychoneuroendocrinology* **135**, 105576 (2021).
4. Cosmin Lazar. imputeLCMD: A collection of methods for left-censored missing data imputation. (2015).
5. Wei, R. *et al.* GSimp: A Gibbs sampler based left-censored missing value imputation approach for metabolomics studies. *PLOS Comput. Biol.* **14**, e1005973 (2018).
6. Bernardoni, F. *et al.* Weight restoration therapy rapidly reverses cortical thinning in anorexia nervosa: A longitudinal study. *NeuroImage* **130**, 214–222 (2016).
7. Hagmann, P. *et al.* White matter maturation reshapes structural connectivity in the late developing human brain. *Proc. Natl. Acad. Sci.* **107**, 19067–19072 (2010).
8. Cammoun, L. *et al.* Mapping the human connectome at multiple scales with diffusion spectrum

MRI. *J. Neurosci. Methods* **203**, 386–397 (2012).

1. Daducci, A. *et al.* The Connectome Mapper: An Open-Source Processing Pipeline to Map

Connectomes with MRI. *PLoS ONE* **7**, e48121 (2012).

1. Van den Heuvel, M. P., Mandl, R. C. W., Stam, C. J., Kahn, R. S. & Hulshoff Pol, H. E. Aberrant Frontal and Temporal Complex Network Structure in Schizophrenia: A Graph Theoretical Analysis. *J. Neurosci.* **30**, 15915–15926 (2010).
2. Carling, K. Resistant outlier rules and the non-Gaussian case. *Comput. Stat. Data Anal.* **33**, 249–

258 (2000).

1. de Reus, M. A. & van den Heuvel, M. P. Estimating false positives and negatives in brain networks. *NeuroImage* **70**, 402–409 (2013).
2. De Reus, M. A. & Van Den Heuvel, M. P. Estimating false positives and negatives in brain networks. *NeuroImage* **70**, 402–409 (2013).
3. Benjamini, Y. & Hochberg, Y. Controlling the False Discovery Rate: A Practical and Powerful

Approach to Multiple Testing. *J. R. Stat. Soc. Ser. B Methodol.* **57**, 289–300 (1995).

1. Venables, W. N. & Ripley, B. D. *Modern Applied Statistics with S*. (Springer New York, New York,

NY, 2002). doi:10.1007/978-0-387-21706-2.

1. Ripley, B. *et al.* Functions and datasets to support Venables and Ripley, ‘Modern Applied Statistics with S’ (4th edition, 2002). (2023).
2. R Core Team. R: A language and environment for statistical computing. R Foundation for Statistical

Computing (2023).

1. Alfons, A. & Ates, N. Y. (Robust) Mediation Analysis. (2023).
2. Hagberg, A. A., Schult, D. A. & Swart, P. J. Exploring Network Structure, Dynamics, and Function using NetworkX. in *Proceedings of the 7th Python in Science conference (SciPy 2008)* 11–15 (2008).
